# Perceptions of employers in South Africa on library and information science graduates’ skills, knowledge and competencies on digital scholarship

**DOI:** 10.1016/j.heliyon.2023.e13531

**Published:** 2023-02-04

**Authors:** Philangani Thembinkosi Sibiya, Patrick Ngulube

**Affiliations:** a Department of Information Science, UNISA, South Africa; b School of Interdisciplinary Research and Postgraduate Studies, UNISA, South Africa

**Keywords:** Perceptions of employers, LIS job Market, LIS education, Digital scholarship, South Africa

## Abstract

The Library and Information Science (LIS) profession is ever evolving partly as a result of the effects of the Fourth Industrial Revolution. For instance, new job requirements on digital scholarship have been noted across the LIS profession, especially in the academic and special research council libraries. Based on these changes, employers of LIS graduates expect that LIS graduates from LIS schools possess digital scholarship knowledge and skills as part of their exit attributes. This paper explores the perceptions of employers of LIS graduates on digital scholarship education. A construct from the Gap Service Quality Model was used as the conceptual framework of the study. The paper followed the interpretive philosophical perspective, through employing a qualitative approach to achieve the aim of the study. A multiple case study design using semi-structured interviews was conducted with directors of both academic and special research council libraries. Five directors were selected purposively as their libraries seemed to be on the forefront of digital scholarship initiatives. It was revealed that employers expected LIS qualifications to have content on digital scholarship. The study discovered that employers of digital scholarship librarians expected them to possess knowledge, skills, and competencies on digital scholarship-related activities. The other expectation was that graduates were supposed to be able to navigate digital scholarship activities at a basic level. Employers of digital scholarship librarians also expected graduates to be familiar with research data management and the ecosystem of digital publications. The survey also highlighted certain difficulties LIS employers had when hiring a LIS graduate. The study recommended that LIS schools should recurriculate to include content on digital scholarship. It was also recommended that LIS schools should have short courses on digital scholarship to cater for practising librarians. Librarians must be involved in lifelong learning in order to understand digital scholarship.

## 1 Introduction

Identifying the needs of the customers and conducting general market research is essential for Library and Information Science schools as a service provider. Library and Information Science (LIS) schools have been in the business of offering LIS education to aspiring information professionals for decades. Nonetheless, technology has been the game changer in almost all disciplines. The changes are noted particularly in science, technology, engineering and mathematics (STEM) field. Wexelbaum [1] and ] Ayinde et al. [2], note that institutions of higher education seeking to promote interdisciplinary study and research and to defend the relevance of the humanities and the social sciences, encourage the integration of STEM (computer science in particular) and the humanities [1,3]. The urgency of digital scholarship education in LIS schools is evident by the growing job positions that require digital scholarship skills, knowledge and competences in the job market. There has been a shift in traditional disciplines due to digital humanities and digital practices [4]. Along the same lines, King [5] mentions that, more recently, a partnership is being forged between digital humanities and digital libraries, leading to the demand for digital humanities centres in academic libraries and an increase in the call for digital scholarship librarians. This is evident in scholars and other researchers conducting their research digitally [5]. These scholars are served by librarians produced by LIS schools; therefore, librarians should always be well equipped with referral information and research skills to collaborate with their patrons. Without proper digital scholarship education, it is impossible to render quality services according to the needs of the patrons.

Digital scholarship education also allows for collaboration between librarians and patrons in discussing issues of the profession and sharing relevant information with researchers, faculty members and other library patrons. To emphasise the importance of digital scholarship, Malhan [6], and Adams et al. [7], suggest that librarians should be trained in digital scholarship since there is an emergence in the work environment of the cutting-edge knowledge, ingenuity and new ideas that make a big difference in advancement. However, Malhan [6] notices that there is a constant searching for talent and employing individuals with agility, requisite skills and competencies, inclination for updating, a penchant for learning and performing in an outstanding manner using innovative approaches. Unfortunately, LIS schools are failing to train individuals in digital scholarship [6,8,9] yet, they expect their graduates to excel in the job market. Therefore, to thrive as LIS schools against the counterparts supplying the market with similar skills and knowledge in the Fourth Industrial Revolution (4IR), LIS schools should offer education relating to the demands of the market.

Digital scholarship education is important, as everyone, whether a practising librarian or a student, needs to be trained in this emerging area. Wexelbaum [1] sums it up by stating that LIS schools must expand their role to providing needs-based continuing education for working professionals in order for them to remain relevant in their jobs. Furthermore, Ayinde et al. [6], proposes that LIS schools in developing countries should provide relevant LIS programmes and supporting infrastructure for teaching and learning in order for students to understand the content on digital scholarship. Locally, Masenya et al. [10], suggest that librarians should attend workshops and conferences relating to the preservation of digital resources to establish or polish their skills in order to thrive in the revolving digital environment. This comes as a wake-up call, as most LIS schools are not offering education on digital scholarship, which impacts the quality of LIS graduates in their first jobs.

A study in the South African context on the change of the labour market, with a specific focus on academic or research libraries amidst digital scholarship has been conducted [11]. This study discovered that most library duties have changed as a result of digital scholarship [11]. New sets of skills, knowledge and competencies have been introduced. The current study hopes to identify the expectations of employers of LIS graduates on digital scholarship skills, knowledge and competencies. Academic libraries and special research council libraries were purposively targeted as they are thought to be the fast-revolving libraries, trying to reach the global trends in digital scholarship.

Generally, perceptions of employers in digital scholarship differ from one individual institution to the next [12]. This study was aimed at exploring perceptions of employers from academic and special research council libraries on whether LIS graduates from LIS schools in South African possess digital scholarship skills, knowledge, and competencies.

## 2 Problem statement

Education in general should produce competent graduates who are fit enough to execute their duties upon completion of a course [2]. The primary aim of LIS education in Africa is to produce competent librarians to manage libraries in various African countries [13]. However, this has not been the case with some LIS schools in South Africa. The LIS schools in South Africa are struggling to offer education that carries digital scholarship skills and knowledge [14]. In line with that, Harris-Pierce et al. [9], are of the view that LIS education has been producing candidates that are unfit for the job market. Apparently, many LIS schools in South Africa remain behind in offering digital scholarship programmes and courses [11,15]. Consequently, a lack of provision of digital scholarship programmes and courses could negatively impact on job opportunities for LIS graduates. The literature reviewed highlighted that the failure to provide the necessary courses that offer competencies, skills and knowledge to support digital scholarship could lead to computer or Information Communication Technology (ICT) specialists and software engineers showcasing in the job market and displacing LIS professionals [2,8,9,14]. Furthermore, this can also result in poor provision of library services due to a lack of digital scholarship core skills, knowledge and competencies required in the digital library environment.

As per the provided elaboration, employers of digital scholarship librarians have a better understanding of their requirements. Identifying the expectations of LIS graduates' employers could assist in understanding the status of LIS education and assist in improving LIS education in South Africa. Therefore, as part of a doctoral study that was aimed to investigate education and training of LIS professionals on digital scholarship, this study explored employers' perceptions of digital scholarship librarians’ skills, knowledge and competencies.

The study gathered perceptions of employers based on the following objectives, which were to.

• Identify the expectations of employers on digital scholarship education
• Establish challenges experienced by employers of digital scholarship librarians
• Identify LIS schools offering proper digital scholarship education
• Provide solutions towards LIS education

## 3 Literature review and conceptual framework

The experience of the perspectives is shaped by the experience of the individual librarians in their respective institutions. Parts of the framework by Zeithaml et al. [16], served as the conceptual basis that structured the study. The Gap Service Quality Model informed this study in mining the perceptions of employers of LIS graduates regarding digital scholarship positions. The researchers should mine the perceptions of employers to ensure customer satisfaction or dissatisfaction; in this case, customers are employers of LIS schools' graduates and the LIS graduates as they study in LIS schools [16]. In essence, the model indicates that antecedents of the desired and the predicted service encompass explicit service promises, implicit service promises, word-of-mouth communication and past experiences [16]. These antecedents assisted in mining the perceptions of employers of librarians. Expectations, as indicated by Zeithaml et al. [16], barriers or challenges, opportunities and strategies to overcoming barriers were sought from the directors of digital scholarship sections in both academic and special research council libraries. The Gap Service Quality Model has been used by most studies that explore user satisfaction and perception, for instance Brown et al. [17], who examined the professional service providers services to medical customers. The authors aimed at seeking the perceptions of customers on services offered to them. This model served to accomplish their study's objectives. This model has direct implications for the current study as it is aimed in exploring the satisfaction of LIS employers as far as LIS education was concerned. The constructs from this model aided in structuring the objective of the study which then informed the themes of the literature review and the analysis of the collected data.

The literature review of this study was based on the objectives of the study as informed by the conceptual framework. Opportunities offered by the LIS education to digital scholarship librarians were reviewed from the literature, challenges experienced by employers when employing digital scholarship librarians were provided and strategies to overcome the challenges were also uncovered as part of the literature reviewed.

### 3.1 Expectations of digital scholarship

Opportunities brought by digital scholarship are generally what the employer of LIS graduate would expect an LIS graduate to possess as the skills, knowledge and competencies one would need to prosper in the digital environment. Digital scholarship brings opportunities to the LIS profession, as the expertise evident at the digital scholarship centre permits researchers to deepen their knowledge, refine their work and collaborate with other disciplines [18]. In an era of visualisation, analytics, big data and new forms of online publishing, central spaces can facilitate knowledge creation and transfer by connecting people, data, and technology in a shared collaborative space [19,20]. The implication is that librarians should not be threatened by the fast-growing technologies brought about by 4IR. Rather, they should embrace them and view them as tools to facilitate their responsibilities. Along the same lines, King [5] sustains that in the wake of the 4IR, a partnership between digital humanities and digital libraries has been forged, calling for an increase in digital humanities or scholarship centres in academic libraries. This has resulted in a dire need to employ digital scholarship librarians. Nevertheless, it is not so clear what the relevant skills are that these librarians need to fulfil their roles [5], as this field is contested by Information Technology (IT) specialists and computer science specialists [2,8,9,14,18,21].

Scholars posit that the role of a digital scholarship librarian lies between digital humanities and digital librarianship as broad areas [5,22,23]. Furthermore, King [5] postulates that librarians are expected to acquire digital skills in order for them not to only support digital humanities, instead, they would play a collaborative role in the process and be viewed as equal partners. Although this assertion is factual, for librarians to match this challenge, it is paramount to include lifelong learning training of digital scholarship librarians. This training, in the form of a module, may equip librarians with tools and competences in digital scholarship methods that will provide them with the knowledge of drafting questions and leading a digital humanities project rather than being supporters [18,20].

Various scholars indicate that numerous new digital scholarship jobs have invaded the library space in the dawn of the 4IR, which are duties performed by both digital scholarship librarians and digital humanists [5,20,[24], [25], [26], [27]]. The opportunities brought to the LIS profession, specifically the digital scholarship librarianship, are mainly drawn from both digital humanities and digital librarianship [5]. Therefore, the digital scholarship responsibilities are important, as they are the new opportunities available for digital scholarship librarians. Examples of opportunities available of digital scholarship librarians according to these authors [5,20,[24], [25], [26], [27]] include digital ecosystem, digital curation, digital humanities data, website management, digital publishing, big data, interface knowledge, coding, collaborative work, metadata enhancement and classification schemas, copyright issues, managing institutional repositories, preservation and sustainability, digital resource creation and content creation.

Most of them appear in a study on emerging LIS jobs as per the influence of the 4IR in South Africa [11]. As part of the change in libraries, the technology in the digital scholarship centres offers librarians an opportunity to build on their expertise; such technologies include laser cutters, Virtual Reality (VR) headsets, high-end scanners, visualisation and video walls [27,28]. It is expected of a digital scholarship centre to have a hub for digital humanities, and for experts to respond to questions about data management and data curation services. However, this may not always be the case, as what works for one centre, may not work for another depending on the centre's requirements [27,28]. In most cases, digital scholarship centres particularly require skills in web development, project management, metadata schemas, GIS specialisation, digitisation specialisation, research data management (RDM), intellectual property and open access (OA) textual and numeric data, and data specialisation [27,29]. In essence, Craft [29] stresses that the potential opportunities are different in every digital scholarship centre, as each centre defines its functions differently depending on the needs of the parent organisation.

The application of structured metadata for textual academic research opens discoverability, which enables access to multiple points of subject access, resulting in high citation index [27]. This implies that the visibility of the researcher and the institution will increase, demonstrating that digital scholarship opportunities are beneficial to the institution. Furthermore, scholars are in consensus that digital scholarship enhances the consortium model, which allows for collaboration discovery and comparison of data results [28,30].

### 3.2 Challenges of Digitals scholarship

Digital scholarship brings many opportunities to the library space; however, certain challenges in the fraternity of librarianship were brought about by the emerging skills introduced by the 4IR. Most of these challenges concern resources, as indicated by several scholars in the literature. As summarised by Mutula [31], the challenges in the African context include a shortage of computers, a lack of clarity of online content, poor internet connectivity, struggles in locating information online, too heavy workload and poor formats of presenting online content. These challenges are still relevant, and they affect both LIS schools and libraries. It is a challenge for academics to learn digital scholarship practices within a particular network [32]. This could result in adequate skills transfer to librarians, as academics are trainers of practitioners; therefore, a lack of knowledge in academics results in poorly trained graduates. Most institutions fail to provide relevant infrastructure for the new environment, especially those involved in digital scholarship, as most stakeholders think of infrastructure only as physical things [20]. However, this is not the case with digital scholarship, as it concerns the sharing of information, collaboration and knowledge creation beyond the physical enterprise [20,33]. It could be that there is a lack of awareness by senior management in the university as far as digital scholarship is concerned [34]. A lack of knowledge and skills when employing digital scholarship librarians have been highlighted as one of the major challenges affecting the LIS fraternity. Such challenges have led to employers in the LIS field employing candidates from other fields to execute librarian-based duties [2]. These challenges are experienced by employers of digital scholarship librarians.

### 3.3 Strategies to overcome digital scholarship barriers

The lack of awareness among the stakeholders of digital scholarship seems to be problematic. The training sessions in the form of workshops, on-the-job training, conferences, and informal and formal courses for digital scholarship may assist in awareness and training of digital scholarship librarians [29]. Furthermore, Craft [29] echoes that funding must be provided to support learning in this area for both librarians and graduate students. Upon return from any funded training, the group that attended can conduct workshops for others for the purpose of knowledge transfer. Partnerships with different digital scholarship constituents by librarians could be beneficial. Muthu et al. [35], and Craft [29] strongly commend the idea of education to address the problem of staff, faculty, students and the university management not being aware that the concept of digital scholarship must be in place.

One of the most important strategies to overcome barriers or challenges in digital scholarship is education on the concept of digital scholarship [28]. Higher education institutions must provide relevant infrastructure and education on digital scholarship, and they have to validate the digital scholarship as valued scholarship [20]. It is not feasibly for information centres to entirely take on the responsibility of digital scholarship education. However, constructing a network of collaborators across a campus that have similar interests in leveraging new technologies and research methods to advance scholarship and learning at different institutions could ease the responsibilities [20,28,29].

Infrastructure is another challenge that most institutions experience, as scholars indicate that parent organisations must be willing to provide relevant infrastructure to support digital scholarship [20,29]. A proper infrastructure would equip the digital scholarship services with the strength to be efficient and effective in attracting more users to the centre. Other organisations rely on the already existing infrastructure to support digital scholarship, but this is not optimal as new equipment should be developed for digital scholarship as it is an emerging trend with completely different needs [27]. Attached to infrastructure is the issue of staffing – a proper model for staffing must be adopted to look into the digital scholarship competencies, skills and knowledge. Desired skills for these kinds of jobs are not easily identified as they vary across disciplines; nonetheless, with a proper staffing model it will be possible to identify the relevant candidates. Some policies and legislation are outdated as they were not targeted at the digital scholarship environment [20]. Therefore, many institutions have to rethink these policies and laws to meet the needs of digital scholarship.

## 4 Methodology

An interpretive paradigm was employed in conducting this study, which allowed for an interpretation of concepts as social contructs [36,37]. The perceptions of employers on LIS graduates' skills, knowledge and competencies were interpreted differently by different role players: this qualified the study as being in the interpretive paradigm [[38], [39], [40]]. The study employed a qualitative approach to achieve the purpose of the study in line with the interpretivism paradigm. In qualitative research, open-ended questions are often used and participants are expected to answer those questions using their own words and perspectives rather than being influenced by the researcher's perceptions of life [37,41]. Specifically, a multiple case study design was utilised to study the perceptions of employers on digital scholarship. The study targeted 10 academic and three special research council libraries in South Africa. However, only five of these libraries participated in the study. Four of them were academic libraries and one was a special research council library. This population was deemed sufficient for a qualitative study as the focus of the interviews were on the perceptions of the employers rather than the content of the LIS curriculum. The University of South Africa (UNISA) ethics committee provided an ethical clearance certificate for the study to be conducted. Prior to the data collection, relevant documentation for ethical clearance purposes and requesting permission to collect data were sent to the relevant institution. Informed consent was obtained from the all the study participants to collect data. Semi-structured interviews were used to collect data from directors of digital scholarship sections in both academic and special research council libraries. Interviews were conducted using MS Teams, which were recorded and pinned in the MS Teams application for ease of retrieval. Each interview took approximately 40–60 min. These interviews were later transcribed into transcripts. A framework by Braun et al. [42], was used to analyse data. The ATLAS. ti 9 software assisted in analysing data. The participants of the study, as earlier indicated, were directors of library services in their respective institutions; therefore, as per the anonymity clause, they were allocated code names. In the ATLAS. ti 9 software they were coded as DS D- 1 up to 5, in reference to digital scholarship directors that participated in the study.

## 5 Findings and discussions of the study

**Fig. 1:**
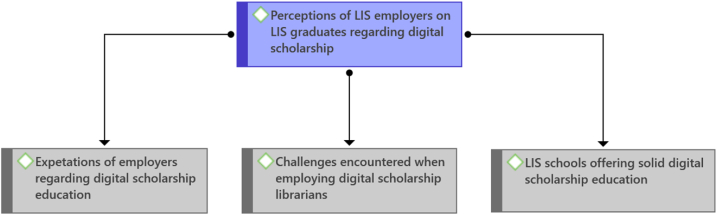
Perceptions of LIS employers on LIS graduates regarding DS.

In the case of this study, directors of academic and special research council libraries were considered to be employers of digital scholarship librarians, as they were the ones who decided on the specifications of a digital scholarship librarian. Based on this theme, three sub-themes emerged, which included expectations of employers regarding digital scholarship competencies, LIS schools offering solid digital scholarship education, and challenges encountered when employing a digital scholarship librarian. These sub-themes were elaborated on in the subheadings below. Fig. 1 displays the relationship of the sub-themes according to the perceptions of LIS employers on LIS graduates regarding digital scholarship.

### 5.1 Expectations of employers regarding digital scholarship competencies

Directors of both academic and research council libraries were asked to share their expectations regarding digital scholarship competencies of LIS graduates. Participants pointed out several competencies that digital scholarship librarians had to possess. Among others, they indicated that LIS graduates must be able to navigate digital scholarship activities at a basic level, should be able to identify the knowledge gaps, and must understand OA, RDM and the publishing ecosystem. In addition to that, the participants suggested that digital scholarship must be embedded throughout the four-year LIS degree. Across all the responses, an understanding of digital scholarship activities was observed by all participants as an important competency among candidates who aspired to be digital scholarship librarians.

Participant DS D-1 elaborated that:

I think library science is still relevant; however, it needs to be coupled with other new programmes to enhance and innovate LIS. Computer competency is critical.

Participant DS D-3 articulated that:

My expectations are that at least a third-year student should be able to know how to go about to navigate digital scholarship activities and to know a little bit more, they don’t have to know everything because of course they will know once they have been employed, but they must have the basic knowledge and be able to identify the gap on what they know.

Participant DS D-4 stated that:

Obviously, I am expecting them to deliver digital scholarship and to build skillsets in that area because that is an imperative area; hence, I don’t believe that it should be a single course where sometimes a student may opt not to take. I believe that all these facets of digital scholarship must be embedded in every course that is taught in LIS schools whether it is introduction to library and information science or metadata, digital scholarship must be there. It must be featured in every course.

Participant DS D-5 postulated that:

LIS schools should produce competent librarians who are able to provide services in predominantly online environments. The Covid-19 pandemic has ushered in a new way of working where online LIS service delivery has become mandatory.

Participant DS D-2 claimed that:

My expectations in terms of my experience, would be a greater understanding of OA which has been fairly limited. I would like to see a publishing ecosystem, so that students are more aware of what goes on the background in terms of how research outputs or articles are generated, a better understanding of RDM in terms of the research data life circle.

The study indicated that employers of digital scholarship librarians expected that they must possess knowledge, skills and competencies in digital scholarship-related activities. It was revealed that graduates should be in a position to navigate digital scholarship activities at a basic level. The study further found that LIS graduates should be able to identify the knowledge gaps and understand the OA. The study also ascertained that, among other expectations of employers from digital scholarship, graduates had to understand RDM and the digital publication ecosystem, and they strongly expected LIS schools to incorporate digital scholarship content through all the levels of the four-year LIS degree.

It is stated by Ayinde et al. [2], that as scholarship was changing, institutions were expected to reshape the skills sets. This applied to the findings of this study, as digital scholarship has emerged and changed most of the ways in which activities were conducted in the library. Echoing the findings of this study, King [5] states that the roles of a traditional librarian had evolved, and this called for a change in the skills sets and training of future librarians in LIS schools. In addition to that digital scholarship librarians must understand the data life cycle management, metadata, RDM, digitisation, digital curatorship and GIS applications in the LIS profession [5]. These findings were in line with those of the current study.

Most participants in this study anticipated digital scholarship to be incorporated into all the levels of the four-year BLIS degree rather than having a separate qualification on digital scholarship. The study further discovered the concurrence among participants that LIS schools should not compromise the core values of librarianship at the expense of technology. This was supported by Hirsh [43], by stating that the inauguration of iSchools resulted in most LIS schools having prioritised technology at the expense of the core values of library science. Along the same lines, the participants of this study cautioned against that trend.

### 5.2 Challenges encountered when employing digital scholarship librarians

Library directors were in an ideal position to point out challenges encountered when employing a digital scholarship librarian. This question was asked to identify these challenges with the hope to later uncover solutions to the identified challenges. Participants provided various challenges encountered when employing a digital scholarship librarian. Among others, the limited pool of candidates for selection, the lack of experience among the candidates and the lack of awareness as far as digital scholarship was concerned were identified. Some even mentioned that they eventually employed people from outside the LIS field due to the lack of candidates.

Participant DS D-1 claimed that:

Librarians do not have technical science skills, for most sciences were never their subjects from basic education right through tertiary education. It then takes a while before an information specialist gets accustomed to science and supporting science researchers in Science, Engineering and Technology disciplines.

Participants DS D-3 revealed that:

Jah well, they are too scared. People are scared about digital scholarship, as I told you that now I have got a digital scholarship librarian, I had only two internal applicants. I think we also had about ten externals, but when we shortlisted, we narrowed it down to two externals, so in totally I had only four as they lacked the skills to be called for interviews. The only candidate that I have employed has a M.IT and PGDLIS which he decided to do like very recently to get a library qualification, but he was very much IT aligned. So yes, there is a fear from people to embrace it, they think it’s too high end, or they will say that “no I am not digital inclined”. There also seems to be a reluctance from even some of the younger generations.

Participant DS D-4 contended that:

Obviously, because I haven’t employed anyone, I won’t know, but what I knew from the on-set is that I am not going to get the person. Especially, if I am looking for an experienced individual because I was reviewing some information from UCT and you find that those who are experienced there will not come to the university like ours. I would rather groom and capacitate my internal team on digital scholarship, so as far back as 2018, I already spotted my team and have sent them to different workshops. It is hard to attract a digital scholarship librarian. I am even trying to get graduates from IT and computer science to fill in this gap as most librarians do not have these digital scholarship related skills. I find the ones from IT and computer science techno-savvy and have high aptitude of technological understanding they even understand metadata and information architecture in a deep way.

Participant DS D-2 indicated that:

So, one of the challenges is, there are not many candidates who are suitable, the pool of candidates is limited, as far as knowledge is concerned, they may be knowledgeable but there would be a lack of experience. One of the things we have come across fairly recently is that places they came from; they were not exposed to broader multiple library functions as we would expect them to be. The problem is that digital scholarship is a very broad field and in terms of its requirements and the manner in which we work as an institution, is such that we work very broadly across the spectrum of the library itself, in other words, all of the library services, including faculty librarianship and beyond, you also work with the technology transfer office to work on the policies for data handling and data management plans and polices for the research office and the library. These things are not taught in most LIS schools, which limit the skills, knowledge and competences. People need to broaden their understanding of what digital scholarship is and that is far beyond the traditional library practices.

The study exposed various challenges that were encountered when employing a digital scholarship librarian in academic and special research council libraries. A limited pool of candidates to choose from, a lack of experience among candidates and unawareness among candidates about digital scholarship were identified as major challenges encountered when employing a digital scholarship librarian. The study discovered that these challenges resulted in the employment of the candidates outside the LIS field, as students from LIS schools were mostly unfit for digital scholarship job positions.

In the extent literature it is noted that there is a lack of skill in digital scholarship [44]. As indicated by Ridge [30] that there is a very small pool of candidates from which to select digital scholarship librarians, as there are few LIS schools that taught digital scholarship, especially in South Africa [15,45]. In agreement with the current study, Craft [29] demonstrated that candidates lack the requirements for digital scholarship vacancies in terms of relevant skills and experience. Generally, there is a lack of basic understanding of digital scholarship among librarians, which is linked to the employment of people from rival disciplines [14,30].

The current study confirmed the findings by Ayinde et al. [2], Harris-Pierceet et al. [9], and Raju [14], that LIS employers would prefer a candidate from other disciplines when it comes to employing a digital scholarship librarian. The failure of the LIS schools to provide the necessary skills on digital scholarship led to ICT specialists and software engineers showcasing in the job market and displacing LIS professionals [9]. This was confirmed by this study, as most of participants indicated that candidates from IT-related disciplines were more knowledgeable than their LIS counterparts as far as digital scholarship was concerned. The challenges experienced by employers of digital scholarship were mainly associated with the curriculum that did not cover digital scholarship, which resulted in librarians being unemployable due to a lack of relevant skills and competencies.

### 5.3 LIS schools offering solid digital scholarship education

Participants were asked to share their views about LIS schools that were offering solid digital scholarship education in their curricula. Surprisingly, only three LIS schools emerged as the ones that had a curriculum in digital scholarship. These schools are the University of Cape Town (UCT), University of Pretoria (UP) and University of Johannesburg (UJ).

Participant DS D-1 identified the following:

UJ, School of Information and Knowledge Management

Although unsure, participant DS D-3 was of the view that:

At the moment, I don’t want to be unfair, but I have only heard of the UCT and UP model they were leading on the RDM and of course, they do library online publishing and they actual won an award about a month or two ago. Perhaps, the other universities do have but I am not familiar, I am just so biased because two of my staff are doing a course with UCT and we have invited the lecturer to come and teach a four-week course to some of my other staff members rather than spending the money paying for logistics to UCT. The course was related to RDM and online publishing.

One participant felt that even though there were two LIS schools that were offering solid digital scholarship curricula, she preferred to groom someone internally. Participant DS D-4 specifically elaborated that:

I haven’t employed anyone for digital scholarship, it is because I know that I might not even get such a person. Well, I am aware of the UCT curriculum, but I would rather get somebody that is very experienced and tech-savvy as enabler of digital scholarship, then I would capacitate that person towards digital scholarship activities. I prefer an internal experienced somebody, instead of a new graduate with less experience possibly half-baked in the digital scholarship area. Even here, I have capacitated someone who have been working at the library for some time and doing master’s in Computer Science. The thing with these new graduates is that it takes time for them to learn and they sometimes lack professionalism. If I were to identify, I would identify UCT and UP graduates.

Participant DS D-2 remarked that:

Not too many of them. If you want names, what I would say? UCT has a postgraduate diploma that focuses on academic libraries. Their courses are essentially postgraduate diplomas, master’s and PhDs with none at undergraduate degree. I have seen that students from those schools in particular have actually done better when it comes in interviewing them and appointing them.

As it was suggested in the literature that benchmarking with sister LIS schools was considered a crucial aspect of curriculum development concerning digital scholarship education [20], this section assists in identifying LIS schools that had a solid digital scholarship focus. The study revealed that there were only three LIS schools that had solid curricula with digital scholarship content, namely UCT, UP and UJ. In South Africa, there are 10 LIS schools that offers LIS education [46]. These schools include UCT, University of Western Cape (UWC), University of Limpopo (UP), University of KwaZulu Natal (UKZN), University of South Africa (UNISA), University of Fort Hare (UFH), Durban University of Technology (DUT), UJ, University of Zululand (UZ) and the UP. However, Mahlatse et al. [45], have noted that only few LIS schools offer education on digital scholarship.

These findings were similar to those of Mahlatse et al. [45], namely that there were only a few LIS schools in South Africa that offered digital scholarship education. These authors further revealed that there were only two LIS schools that offered qualifications in digital scholarship [45]. The current study did not agree with Mahlatse et al. [45], in the sense that participants identified UJ as one of the LIS schools that offered digital scholarship. Along the same lines, Wood et al. [15], also underscored that South African LIS schools were struggling to offer the digital scholarship skills and knowledge needed in the digital environment. The implication was that LIS schools without digital scholarship content could collaborate and benchmark with the LIS schools that had content on digital scholarship. LIS schools need to update their curricula to include digital scholarship content [14]. This study discovered that other LIS schools were already revising their curricula to include digital scholarship content.

A degree in data science was offered at the Sol Plaatje University and no LIS school was offering content on this aspect of digital scholarship [45]. This is supported by Tu et al. [47], who state that most digital scholarship contents emanated from computer science and IT. Therefore, the assertion that there were few LIS schools teaching digital scholarship may be the result of a lack of expertise among LIS academics. This may change in future, as the study discovered that LIS schools were in the process of recurriculating to include digital scholarship content. It is proposed by Koehl et al. [44], that even librarians already practising need to be taught digital scholarship, as the demand from the job market was increasing as far as digital scholarship was concerned, yet the skill set was lacking.

## 6 Conclusions

The conclusions of the study are based on the findings of the literature in addition to the findings of the study. Ultimately, it was discovered from this study that LIS employers expected LIS graduates to have knowledge, skills and competencies on digital scholarship. Although this was the expectation, it was noted that few LIS schools had content on digital scholarship in South Africa. The institutions were UCT, UP and UJ, as disclosed by the participants. As a result, there was a limited number of LIS graduates that could fit into digital scholarship positions in academic and special research council libraries in South Africa. Most employers indicated that they had no confidence in LIS schools’ graduates as most of them demonstrated no understanding of digital scholarship units within the library, as compared to their IT and computer science counterparts.

It was clear from the study that employers of LIS graduates preferred to employ candidates from other related fields instead of LIS schools’ graduates when it comes to digital scholarship positions. This is a wakeup call for the LIS schools to question their curricula and properly channel it towards the requirements of the employer. Failure to do so will exacerbate the notion of employing candidates from other fields in positions of LIS professionals. This study is limited in sense that it reports on the findings from the employers of digital scholarship librarians not the entire population of the doctoral study. Nonetheless, it was noted from the doctoral thesis and the extent literature (cited in the) that very few LIS schools taught digital scholarship contents in their curricula in South Africa. Nevertheless, data from the content analysis and the lecturers was not reported in this study as it is beyond the scope of this study it forms part of the other publication.

## 7 Recommendations

This section provides recommendations as far as employers of digital scholarship librarians in both academic and special research council libraries are concerned. The recommendations are also based on the findings of the literature. The findings of the study indicated that for LIS graduates to be employed in digital scholarship sections, they must possess knowledge, skills and competencies in digital scholarship. These expectations are mainly from LIS schools, as they are the primary trainers of librarians in the country. Therefore, it is expected that the existing outcomes of LIS qualifications should include the required knowledge, skills and competencies in digital scholarship.

The study indicated that there were only three LIS schools that offered digital scholarship education in South Africa. In accordance with these findings, it is recommended that other LIS schools in the country must recurriculate and include the digital scholarship content in their curricula to increase the scope of the pool of the candidates applying for digital scholarship jobs. As a result of a lack of the candidates that are fit for digital scholarship job positions, employers of LIS graduates preferred to employ candidates from other IT-related fields instead of LIS professionals. Therefore, there is an urgent need for the LIS schools to include digital scholarship content in the LIS curriculum. It is also recommended that the LIS employers should employ LIS graduates in digital scholarship positions, even though they lacked relevant knowledge and skills. On-the-job training can be adopted to ensure skills transfer. LIS schools must ensure that they have short learning programmes on digital scholarship for already practising librarians to acquire the relevant skills and knowledge on digital scholarship.

## Author contribution statement

Philangani Thembinkosi Sibiya: Conceived and designed the experiments; Performed the experiments; Analyzed and interpreted the data; Contributed reagents, materials, analysis tools or data; Wrote the paper.

Patrick Ngulube: Conceived and designed the experiments; Performed the experiments; Analyzed and interpreted the data; Contributed reagents, materials, analysis tools or data; Wrote the paper.

## Funding statement

Dr Philangani Sibiya was supported by 10.13039/100007431NRF [SFH170526233651].

## Data availability statement

The researcher is still in the process of deposing the data into the University of South Africa Library's institutional repository. The data will soon be available for the public.

## Declaration of interest's statement

The authors declare that they have no known competing financial interests or personal relationships that could have appeared to influence the work reported in this paper.
